# The Creative Neurons

**DOI:** 10.3389/fpsyg.2021.765926

**Published:** 2021-11-22

**Authors:** Mark V. Flinn

**Affiliations:** Department of Anthropology, Baylor University, Waco, TX, United States

**Keywords:** creativity, innovation, evolution, family, culture

## Abstract

Creativity generates novel solutions to tasks by processing information. Imagination and mental representations are part of the creative process; we can mull over ideas of our own making, and construct algorithms or scenarios from them. Social scenario-building can be viewed as a human cognitive “super-power” that involves abstraction, meta-representation, time-travel, and directed imaginative thought. We humans have a “theater in our minds” to play out a near-infinite array of social strategies and contingencies. Here we propose an integrative model for why and how humans evolved extraordinary creative abilities. We posit that a key aspect of hominin evolution involved relatively open and fluid social relationships among communities, enabled by a unique extended family structure similar to that of contemporary hunter-gatherer band societies. Intercommunity relationships facilitated the rapid flow of information—“Culture”—that underpinned arms-races in information processing, language, imagination, and creativity that distinguishes humans from other species.

## Introduction: Origins

Novelty is risky because the outcomes of untried behaviors may be difficult to predict. Unbridled changes, like mutations, usually have deleterious results as they stray from proven recipes. Hence organisms have evolved constraints on innovation (Lefebvre et al., 2004; Brosnan and Hopper, 2014), and learned transmission biases (Campbell, 1960; Shettleworth, 1998). Our hominin ancestors, however, did something extraordinary: they evolved cognitive aptitudes that underpin a system of cumulative culture that generates rates of innovation that are orders of magnitude faster and more efficient than other species (Tomasello, 1999, 2016; Whiten and Erdal, 2012; Legare and Nielsen, 2015; Fuentes, 2017; Wadley, 2021; cf. Bandini and Harrison, 2020). The puzzle is, why humans? Why not chimpanzees or bonobos or elephants or dolphins or ants? Why are humans alone the “hyper-cultural” species? What were the selective pressures that among all God’s creatures pushed our hominin ancestors so rapidly and extensively down the evolutionary pathway of innovative and cumulative culture?

Humans (*H. sapiens*) have big (∼1,300cc) brains, walk upright, use projectile weapons, do not have outward cues of ovulation, have menopause, and an extended period of child development. This suite of characteristics distinguishes us from our primate relatives and may provide important insights into hominin evolution (Alexander, 1990b; Chapais, 2008; Milks et al., 2019). An additional critical human trait is the expanded family. Hominins increasingly diverged from our hominoid relatives in paternal, sibling, grandparental, mating, and affinal relationships (Hrdy, 1981, 2009; Chapais, 2008; Hawkes, 2020), although the timing is uncertain (Duda and Zrzavý, 2013). Human brothers and sisters maintain life-long connections across residential distance. Affines (e.g., “in-laws”) and other non- or distant kin cooperate in complex ways (e.g., Macfarlan et al., 2014; Chagnon et al., 2017). Grandparents link multiple generations. These relationships facilitate the flexible “nested-coalitions” structure of human societies, connecting individuals in different communities and hence creating opportunities for cultural transmission among and within groups at warp speed.

## Family and Sociality

“Why are we all alone [in]… our tendency and ability to cooperate and compete in social groups of millions?” Alexander (1990b, p. 1)

“… our foraging ancestors evolved a novel social structure that emphasized bilateral kin associations, frequent brother-sister affiliation, important affinal alliances, and coresidence with many unrelated individuals.” Hill et al. (2011, p. 1289)

Human sociality is remarkable in many respects. Our potential for flexible coalitions and alliances is exceptional (e.g., Chagnon, 1988; Choi and Bowles, 2007; cf. Fragaszy and Visalberghi, 1990; Bissonnette et al., 2015; Vale et al., 2021), and has deep evolutionary roots (Wrangham, 1999; Leblanc, 2003; Churchill et al., 2009; Feldblum et al., 2021). The origins are posited to involve unusual aspects of human family relationships—stable breeding bonds and fathering, brother-sister bonds, grandparenting, bilateral kin bonds, affinal bonds—that facilitate interaction among individuals residing in different groups and thereby kindle cumulative culture (Alexander, 1979; Hrdy, 1981; Chapais, 2008; Flinn, 2017). These relationships are underpinned by evolved human neurobiological and neuroendocrinological mechanisms (MacDonald and MacDonald, 2010; Rilling and Mascaro, 2017; Stout and Hecht, 2017; Habecker and Flinn, 2019) but are flexible and diverse (e.g., Walker et al., 2010). Studies of social networks in hunter-gatherer bands are consistent with this family sociality link (Hill et al., 2011; Walker et al., 2011, 2013; Migliano et al., 2017, 2020), and appear to have a long prehistory (Mcbrearty and Brooks, 2000; Coward and Grove, 2011; Sikora et al., 2017; Brooks et al., 2018; Lombard and Högberg, 2021).

## Culture and Imagination

The social world is a rich source of useful information for cognitive development. The human brain appears designed by natural selection to acquire and use information from other minds (Flinn, 1997; Bjorklund and Pellegrini, 2002; Adolphs, 2003; Sterelny, 2012). Transmission via social learning might seem to enable “culture” having its own evolutionary system with separate inheritance mechanisms (for reviews, see Dawkins, 1982; Laland et al., 2000; Henrich and McElreath, 2003; Richerson and Boyd, 2005). Other perspectives emphasize the biology of learning (e.g., Heyes and Galef, 1996; Shettleworth, 1998; Tomasello, 1999; Galef, 2004), where “culture” is viewed as an aspect of phenotypic plasticity (Flinn and Alexander, 1982; Alexander, 2006).

My intent here is to expand the evolutionary perspective of culture beyond the concepts of “dual-inheritance” or of “evoked culture” as behavioral responses to variable environments influenced by task-specific psychological modules. I suggest that evolutionary developmental biology and its reemphasis of the complexity of ontogeny (West-Eberhard, 2003) may provide important insights into culture and its creative variants (Alexander, 1990a; Heyes and Frith, 2014).

Phenotypic variation involves genetic differences and ontogenetic responses to the environment (Schlichting and Pigliucci, 1998; Gottlieb, 2002; West-Eberhard, 2003). Learning biases involve similar “reaction norms” (Heyes and Galef, 1996). Cultural variations have added complexities for inferring evolutionary design. The “creative neurons” and cumulative culture expand human behavior in extraordinary and unique ways, including the arts, literature, spiritual beliefs, technology, and complex sociality (Carroll, 2013; Muthukrishna and Henrich, 2016; Dubourg and Baumard, 2021).

Culture may be viewed as a dynamic information pool that coevolved with intelligence, including social learning aptitudes and language (Flinn, 1997; Sterelny, 2007; Pagel, 2012; Legare and Nielsen, 2015). As the power of information in hominin social interaction increased, culture and tradition were critical for social cooperation and competition (Coe, 2003; Sternberg and Grigorenko, 2004; Baumeister, 2005; Birch and Heyes, 2021). Displays of creativity, if appreciated and rewarded, can be avenues to social success.

Innovation is key. Without new ideas, cumulative culture has nowhere to go. Imagination and creativity fuel the informational arms race that underlies culture. Static reaction norms that influence evoked culture within specific domains (Tooby and Cosmides, 1992; Buss, 1995) are useful but insufficient. The human mind is not constrained to a predetermined Pleistocene set of options (Rogers, 1988). Creative culture goes beyond simple constrained phenotypic plasticity; the constraints must contend with novelties generated from collective imaginations. The human jukebox advances beyond an old selection of tunes; the Beatles displaced Elvis who borrowed from the Blues (see Wood et al., 2021). In some domains, better mousetraps keep beating last year’s models.

Keeping pace in hominin red queen social competition involves imitation of success. Leading the pack requires creativity to produce new solutions to beat the current winning strategies. Informational “mutations,” however, are risky; hence the increasing advantage of cognitive abilities that could refine choices among imagined innovations in dynamic, complex social scenarios (Liberman and Trope, 2008). The theater of the mind that allows humans to “understand other persons as intentional agents” (Tomasello, 1999, p. 526) is a testing ground for evaluation and refinement of creative solutions to the never-ending novelty of social arms races. Selecting the potential winners from novel information generated by the creative mind likely involves cognitive mechanisms for recursive pattern recognition in the open domains of language (Deacon, 1997; Nowak et al., 2001), social dynamics (Flinn and Ward, 2005; Geary, 2005; Shipton, 2019), and technology (Osiurak and Reynaud, 2019). The evolutionary basis for these skills underlying cumulative culture involves a process of “runaway social selection” (Flinn and Alexander, 2007).

## Imagination and Runaway Social Selection

Darwin (1871) distinguished between (a) selection from environmental factors such as predators, climate, and food, and (b) selection from interactions among conspecifics (i.e., competition among members of the same species for resources such as nest sites, food, and mates). We may consider the former *natural selection* and the latter *social selection*—sexual selection a special subtype (West-Eberhard, 1983). Evolutionary changes resulting from these two types of selection—natural and social—are often different in significant ways (West-Eberhard, 2003; Alexander, 2006).

Natural selection resulting from interactions between species, for example with pathogen–host red queen evolution (Hamilton et al., 1990), can be intense and ongoing. A significant portion of genetic changes in human evolution occurred in response to infectious disease (Karlsson et al., 2014). Intraspecific social competition may also cause rapid evolutionary changes (Hamilton, 1970; Connell, 1983). A significant portion of genetic changes in human evolution involved changes in the brain (Preuss, 2012; Wei et al., 2019). Reduced constraints from natural selection (predators, climate, foraging, …), in combination with increased social competition, can result in a runaway process. Human evolution evidences such conditions (Flinn et al., 2005; Alexander, 2006). Hominins increasingly became their own potent selective pressure via social competition involving coalitions (Alexander, 1990b; Wrangham, 1999; Geary and Flinn, 2001, 2002; Leblanc, 2003; Choi and Bowles, 2007; Summers et al., 2020; e.g., Chagnon, 1988; Flinn et al., 2012) and control of their ecologies via niche construction (Deacon, 1997; Odling-Smee et al., 2013). In combination with changes in population density, mobility, and opportunities for exploitation of new environments, the push for information was on.

The most exceptional human mental aptitudes—language, imagination, self-awareness, Theory of Mind (ToM), foresight, mental time travel, and consciousness—involve social relationships (Dunbar, 1997; Siegal and Varley, 2002; Tulving, 2002; Adolphs, 2003; Heyes, 2003; Geary, 2005; Suddendorf et al., 2009). Human sociality involves multiple-party reciprocity and shifting nested sub-coalitions that have high information-processing demands for the cognitive mechanisms that underlie social competency. Social competition within and among hominin communities had increasing amounts of novel information and creative strategies. Creative culture and social-scenario building were increasingly important selective pressures on the evolving brain. Social cleverness enhanced by the powers of imagination could subsequently catalyze aesthetics, beliefs in the supernatural, fictional storytelling, and technological innovations.

## Evolution of the Creative Cultural Brain

The human brain has high metabolic costs (Kuzawa et al., 2014), takes years to develop (Leigh, 2004), evolved rapidly (Lee and Wolpoff, 2003; Bruner, 2021), enables behavior to change quickly, and generates high levels of novel information. We have posited that its primary functions include engaging with other human brains (Alexander, 1989; Adolphs, 2003; Gallagher and Frith, 2003; Roth and Dicke, 2005; Dunbar, 2020). Success is achieved not by strength, foot-speed or antibody production but by data processing in the social mind.

Some of the least explored frontiers of creativity from the Tinbergen (1963) perspective (for review of Tinbergen’s integration of questions from development, adaptive function, mechanisms, and evolutionary history, see Pfaffa et al., 2019) are the neurobiological and neuroendocrinological mechanisms that underpin our imaginations (Jung et al., 2013). It seems unlikely that there are singular “creative neurons” or even localized modules for imagination; these abilities instead result from complex systems involving interaction among many parts of the brain (Semendeferi et al., 2001; Herrmann et al., 2007; Fink et al., 2009; Dean et al., 2013; Sherwood and Gómez-Robles, 2017; Bruner, 2021). Some of these “social parts” of the human brain that are different from our primate relatives are asymmetrically localized in the prefrontal cortex, particularly the dorsolateral prefrontal cortex and frontal pole, but connectivity among other areas such as the hippocampus also appear relevant (Allman et al., 2001; Semendeferi et al., 2001; Rilling et al., 2002; Bzdok et al., 2016; Sherwood and Gómez-Robles, 2017; Beaty et al., 2018; Ardesch et al., 2019; Barry et al., 2019; Cabeza et al., 2020; Bruner, 2021; Parelman et al., 2021; for review, see Geary, 2005; Jung et al., 2010). These parts of the human brain enable “social scenario building” or the ability to “see ourselves as others see us so that we may cause competitive others to see us as we wish them to” (Alexander, 1990b, p. 7) and several odd cognitive abilities such as understanding sarcasm (Shamay-Tsoory et al., 2005), romantic love (Bartels and Zeki, 2004), and morality (Moll et al., 2005). Other mechanisms that are involved in linking family relationships to an open and creative learning environment for the human child include affiliative neuropeptides, dopamine reward circuits, and the hypothalamic-anterior pituitary-adrenal system (Gimpl and Fahrenholz, 2001; Flinn, 2006; Gordon et al., 2010; MacDonald and MacDonald, 2010; Flinn et al., 2011; Habecker and Flinn, 2019, 2021; Quintana et al., 2019; Ponzi et al., 2020; Chong et al., 2021; Grinevich and Neumann, 2021).

Human life history, including the special stage of childhood, facilitates development of important social skills (Joffe, 1997; Flinn and Ward, 2005; Muehlenbein and Flinn, 2011; Sterelny, 2012; Legare, 2017), and creativity more generally. Learning, imagination, and experience are useful for developing social skills. Chronic isolation and solitude are usually unpleasant and can inhibit development of social skills and friendship networks (Bzdok and Dunbar, 2020).

## Evolution of the Creative Child

The human infant needs a protective environment provided by intense parental and alloparental care in the context of extended kin groups and communities (Chisholm, 1999; Belsky, 2005; Hrdy, 2005; Flinn and Leone, 2006). The human baby is physically altricial. The infant delays investing in locomotion, defense, and food acquisition systems, instead working on mental skills such as language that produce a socially competent adult phenotype (Alexander, 1987, 1990a; Flinn, 2004). The brain grows quickly, focusing learning on the social environment (Bloom, 2000; Geary and Bjorklund, 2000; Bjorklund and Pellegrini, 2002; Geary and Huffman, 2002). The information-transmission enabled by linguistic competency provides access to the knowledge available in other human minds. Communication via language facilitates social dynamics of human communities (Dunbar, 1997; Corballis, 2017) and accelerates cumulative culture. Creativity and spread of innovations is enabled by recursion and symbolic representation in human language.

## Predicting the Pathways of Informational Novelty

Humans generate extraordinary levels of novelty by cognitive processing of abstract mental representations. Imaginative human minds navigate a dynamic information environment of their own creation. The method to the madness is that we produce new ideas built on the old. Human culture is cumulative. Most creations, however, are flashes in the pan, fleeting experiments that did not catch on. What accumulates is part chance, and part filtering by a multitude of minds designed by selection to make good choices.

The apparent mayhem of cultural information may result in part because it is a fundamental aspect of human social coalitions. Seemingly arbitrary shifts in cultural traits—music, art, perceptions of beauty, flags, clothing styles, recipes, dialects—may involve information “arms races” and our special sensitivity to status and social context (e.g., Boyer, 1998; Carruthers, 2002; Sperber and Hirschfeld, 2004; De Dreu et al., 2010; Cabeza et al., 2020). Culture is contested because it is a contest worth winning.

Conformity and imitation of leaders and other high status models may push culture in directions contrary to predictions from simple functional concerns or evolved psychological mechanisms. Hence the apparent lack of a simple biological utilitarianism of culture and the significance of historical context and social power (Wolf, 2001). Deconstruction—analysis of meaning of the words and actions of others—is a complicated but necessary enterprise, for we are all players in the social arena trying to outmaneuver one another, but often by prosocial tactics. We are evolved participants cooperating and competing in a dynamic, creative cultural context (Kenrick et al., 2003; Sherwood and Gómez-Robles, 2017); our imaginations pushing the envelopes of phenotypic plasticity.

## Data Availability Statement

The original contributions presented in the study are included in the article/supplementary material, further inquiries can be directed to the corresponding author/s.

## Author Contributions

The author confirms being the sole contributor of this work and has approved it for publication.

## Conflict of Interest

The author declares that the research was conducted in the absence of any commercial or financial relationships that could be construed as a potential conflict of interest.

## Publisher’s Note

All claims expressed in this article are solely those of the authors and do not necessarily represent those of their affiliated organizations, or those of the publisher, the editors and the reviewers. Any product that may be evaluated in this article, or claim that may be made by its manufacturer, is not guaranteed or endorsed by the publisher.
